# Research progress on microcirculatory disorders in septic shock: A narrative review

**DOI:** 10.1097/MD.0000000000037273

**Published:** 2024-02-23

**Authors:** Hui Wang, Hong Ding, Zi-Yan Wang, Kun Zhang

**Affiliations:** a Department of Intensive Care, Affiliated Hospital of Chengde Medical University, Chengde, China.

**Keywords:** critical care, hemodynamic monitoring, microcirculation, sepsis, septic shock, tissue perfusion

## Abstract

Hemodynamic coherence plays a critical role in the outcomes of septic shock. Due to the potential negative consequences of microcirculatory disorders on organ failure and clinical outcomes, the maintenance of a balance between the macrocirculation and microcirculation is a topic of significant research focus. Although physical methods and specialized imaging techniques are used in clinical practice to assess microcirculation, the use of monitoring devices is not widespread. The integration of microcirculation research tools into clinical practice poses a significant challenge for the future. Consequently, this review aims to evaluate the impact of septic shock on the microcirculation, the methods used to monitor the microcirculation and highlight the importance of microcirculation in the treatment of critically ill patients. In addition, it proposes an evaluation framework that integrates microcirculation monitoring with macrocirculatory parameters. The optimal approach should encompass dynamic, multiparametric, individualized, and continuous monitoring of both the macrocirculation and microcirculation, particularly in cases of hemodynamic separation.

## 1. Introduction

Sepsis is defined as an infection-induced host imbalance response leading to life-threatening organ dysfunction. As a common disease in critically ill patients, septic shock is characterized by high mortality, treatment costs, and morbidity. The in-hospital case-fatality rate of sepsis is more than 40%.^[1,2]^ The early identification and control of the infection, as well as the optimal management of hemodynamics, are crucial in the clinical management of septic patients.

In recent years, the interplay between macrocirculation and microcirculation has garnered growing attention in the management of septic shock. Numerous studies have revealed that even though resuscitation efforts can restore normal systemic hemodynamic parameters, microcirculatory disturbances may persist. Inadequate resuscitation measures may fail to restore the microcirculation and often result in tissue edema, increased diffusion distance between capillaries and tissue cells, and, ultimately, poor tissue perfusion and oxygenation.^[3]^ However, in the early stages of septic shock, it can be difficult to assess the status of the microcirculation and thus achieve concordance between the macrocirculation and microcirculation. This narrative review synthesizes research on microcirculatory disorders associated with sepsis, summarizing the impact of septic shock on microcirculation and the methods for its monitoring. It highlights the importance of microcirculation in treating critically ill patients and proposed a referable evaluation framework to enhance the comprehension of the management strategy that integrates microcirculation monitoring with macrocirculation parameters.

## 2. Methods

An extensive search was conducted in the PubMed, Web of Science, and CNKI databases for articles published between 2001 and 2023. Keywords used included “sepsis,” “septic shock,” “tissue perfusion,” “microcirculation disorders,” “critical care,” “hemodynamics,” and “treatment.” The search included reviews, case reports, clinical trials, observational studies, and animal studies, with no restrictions on publications, journals, or languages. Utilizing the SANRA Narrative Review Article Quality Assessment Scale, 2 reviewers (K.Z. and H.W.) screened abstracts or full texts to identify relevant research on the progression of sepsis-related microcirculation. Disagreements were resolved through discussion. All selected articles were thoroughly reviewed, and references cited within these articles were also examined. After a thorough literature search and screening, 78 literatures from 2001 to 2023 were finally used in this review, and the data were organized to summarize the research progress of microcirculation related to septic shock. Specifically, there were 13 literatures on “microcirculation structure,” 15 literatures on “mechanism of microcirculation disorders,” 30 literatures on “methods of microcirculation monitoring,” and 20 literatures on “significance of microcirculation.” Given the nature of this review as a literature analysis, ethical approval was not necessary.

## 3. Discussion

### 3.1. Structure and function of the microcirculation

**Figure 1.**
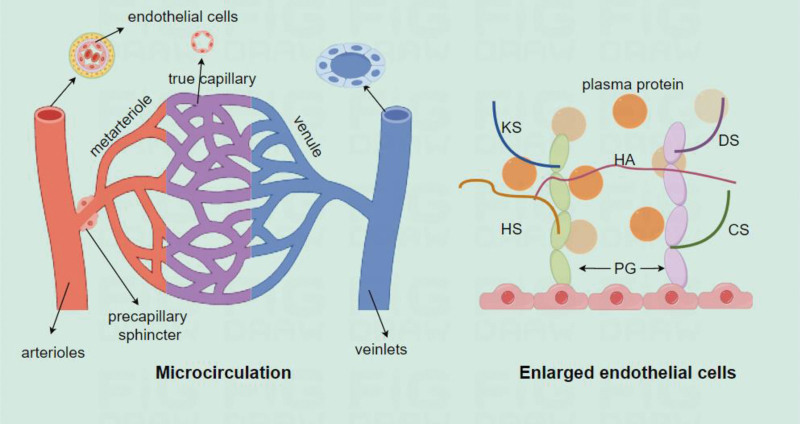
Left: Microcirculation structure and function. The microvascular network consists of arterioles, capillaries, venules and veinlets. Capillaries have only 1 layer of endothelial cells, providing a place for the exchange of gases, nutrients, and metabolic products. Right: Enlarged the endothelial cells. The glycocalyx consists of PGs, GAGs, membrane glycoproteins, and associated plasma proteins. GAG side chains are connected to PGs, forming the structural framework of the glycocalyx. The 5 most prevalent types of GAG chains are HS, CS, HA, DS, and KS. The glycocalyx functions as a barrier to regulate the permeability of the vascular endothelium, and plays a role in maintaining microcirculation homeostasis and perfusion (by Figdraw). CS = chondroitin sulfate, DS = dermatostatin sulfate, GAGs = glycosaminoglycans, HA = hyaluronic acid, HS = acetylheparin sulfate, KS = keratostatin sulfate, PGs = proteoglycans.

The microcirculation is composed of a network of microvascular structures, endothelial cells, pericytes, smooth muscle cells, red and white blood cells, platelets, and lymphatic vessels, collectively facilitating essential functions such as nutrient exchange, waste removal, immune responses, and blood flow regulation within tissues.^[4,5]^ In healthy adults, the microcirculation accounts for approximately 10 percent of the overall circulating blood volume (Fig. 1).

The microvascular network consists of arterioles, capillaries, venules and veinlets with a diameter ranging from 5 to 100 µm. The walls of the arterioles are made up of endothelial cells and a thin layer of smooth muscle cells, which are highly myotropic and have strong contraction and diastole capacity. The arterioles can divide into smaller branches until they reach the level of capillaries.^[6]^ The capillaries have only 1 layer of endothelial cells, lack muscular tissue and are attached to a basement membrane. The venules are composed of intermittent smooth muscle bands. The venules collect blood from capillaries and serve as the transition between the microcirculation and larger veins. The small arteries and capillaries are continuously subjected to high pressure from the macrocirculatory system.^[7,8]^

The endothelial surface layer plays an important role in the regulation of blood flow within the microcirculatory system and the preservation of microvascular integrity and function. The endothelial surface layer is covered by a gel-like layer known as the “glycocalyx” or the “polysaccharide envelope.” This layer occupies 25 percent of the vascular space. Under an electron microscope, this surface appears as a light blue fluorescent substance of about 20 nm. The glycocalyx is mainly composed of proteoglycans, glycosaminoglycans (GAGs), membrane glycoproteins, and plasma proteins. The proteoglycans and GAGs form the backbone of the glycocalyx.^[9,10]^ The glycocalyx protects the sensitive vascular endothelium and regulates the vascular fluid shear. In addition, it also assists in mechanical stimulation, which leads to the activation of endogenous carbon monoxide synthase, which in turn initiates a series of cascade reactions that regulate vasodilation and improve vascular permeability. Meanwhile, the glycocalyx also has anticoagulant and anti-adhesion effects, which can inhibit microvascular thrombosis, regulate the adhesion of leukocytes on the endothelium, and attenuate the oxidative stress of endothelial cells necessary to control the damage of local inflammatory mediators on endothelial cells. The glycocalyx acts as a permeability barrier and is essential for the regulation of microcirculatory homeostasis and perfusion.^[11]^

Jung et al^[12]^ showed that a high serum level of mucin glycan-1 is indicative of glycocalyx shedding and could be used as a predictive marker for early short-term mortality in acute coronary syndromes. Studies^[13,14]^ have also demonstrated that the glycocalyx has an important role in the maintenance of vascular homeostasis and the development of coronary system disorders. Moreover, endothelial glycocalyx (eGC) damage was correlated with the occurrence and development of coronary atherosclerosis.^[15]^

The microcirculation is in direct contact with parenchymal cells and adapts to parenchymal cell dynamics to facilitate the exchange of solutes between vessels and tissue cells. Recent studies have shown that tissue oxygenation occurs mainly at the end of small arteries, while the removal of metabolites occurs mainly at the capillary level.^[8,16]^ Healthy microcirculation function is the basis for ensuring that the body organs are in a normal perfusion state. However, the microcirculation can be altered by various pathological conditions, including bacterial and viral infection, trauma, edema, septic shock, severe inflammatory reaction, and organ transplantation rejection. Failure to restore the microcirculation can lead to multi-organ failure.

### 3.2. Mechanisms of microcirculation disorders

The development of microcirculatory disorders involves 3 steps: inadequate organ perfusion, development of a systemic inflammatory response, and eventually organ failure. According to research findings, sepsis has the potential to disrupt the innate regulation of the microcirculatory system through a range of mechanisms, including endothelial cell damage, increased vascular permeability, the release of vasoactive and inflammatory factors, impairment of the glycocalyx, alterations to circulatory cells, and the formation of microthrombi.^[17]^ The damage caused to the microcirculatory system is often not reversible, even after volume resuscitation.

#### 3.2.1. Endothelial cell damage.

The vascular endothelial cells are surrounded by a semipermeable membrane that acts as a barrier between the blood and the vascular smooth muscle cells. The cell membrane also regulates the vascular tone, inhibits the inflammatory response, and prevents the formation of thrombi.^[18]^ As a consequence, injury to endothelial cells can lead to significant structural damage to the microvascular system and disrupt the delicate hemostatic balance of the system, particularly during the acute phase of septic shock. Once damaged, the endothelial cells are shed, and their loss weakens the vascular structure and promotes the release of vasodilatory and vasoconstrictive factors. The release of these substances also triggers a cytokine storm, which is characterized by the release of large amounts of inflammatory factors and increased capillary permeability.^[19]^ The impact of endothelial cell damage in the development and progression of microcirculatory disorders has been confirmed by various studies. Johansen et al^[20]^ found that the breakdown of endothelial cells triggers the release of syndecan-1 and soluble thrombomodulin glycoproteins. As a result, these glycoproteins could be used as predictive markers for endothelial injury, multi-organ failure, and 90-day mortality in sepsis patients. Li et al^[21]^ found in an early cecal ligation and puncture-induced sepsis mouse model that the disruption of bone marrow kinase on the X chromosome leads to increased thrombin-mediated permeability in endothelial cells. This phenomenon was not detected in other cell types, such as leukocytes and platelets, suggesting that endothelial dysfunction plays a role in the regulation of vascular permeability and the development of sepsis.

#### 3.2.2. The degradation of the eGC.

Studies have also found a high correlation between damage to the eGC and microcirculatory disturbances due to sepsis. The eGC is known to contain 5 types of GAG chains, namely, acetylheparin sulfate, chondroitin sulfate, hyaluronic acid, dermatostatin sulfate and keratostatin sulfate. The degradation of the endothelial lining caused by sepsis can trigger the release of these GAG chains into the bloodstream.^[22,23]^ Furthermore, during sepsis, the release of inflammatory factors initiates the activation of degradative enzymes, including metalloproteinases, acetylheparinase, and hyaluronidase. These enzymes contribute to the progressive degradation of the glycocalyx layer.^[24]^ Consequently, this process results in leukocyte adherence and capillary leakage, allowing plasma proteins and fluids to infiltrate the third interstitial space, thereby triggering tissue edema.^[25]^ Simultaneously, glycocalyx degradation can stimulate neutrophil aggregation through alternate pathways, further intensifying the inflammatory response and disease progression.

Numerous studies have indicated that the degradation of the glycocalyx can serve as a valuable biomarker for assessing microcirculatory damage.^[26]^ Rovas et al^[27]^ conducted a study in which they investigated the reverse parameter perfusion boundary region of eGC size beneath the tongue. The researchers observed that sepsis patients exhibited lower microcirculation parameters, specifically the mean flow index and perfused vessel proportion, compared to a group of healthy individuals. Furthermore, the perfusion boundary region value in sepsis patients was significantly higher than that of the healthy control group, suggesting impaired glycocalyx integrity. In a mouse model study of lipopolysaccharide (LPS) induced lung injury, a notable increase in acetylheparinase activity was observed within the pulmonary vascular endothelium. The increased enzymatic activity subsequently led to the detachment and thinning of the eGC. However, the preadministration of the serine protease inhibitor ulinastatin significantly attenuated the LPS damage and reduced the degree of lung injury in mice.^[28]^ Lygizos et al^[29]^ found that the activation of glomerular acetylheparanase was closely related to the loss of glomerular filtration rate in cecal ligation and puncture mouse model. The degradation activity of this enzyme could be detected in the urine and reached its highest value 4 hours after tissue injury and returned to normal levels within 24 hours. These results indicate that in sepsis-induced renal injury, glomerular acetylheparinase activation occurs at an early stage. However, glycocalyx damage can vary in different organs. Several studies have suggested that patients with nonpulmonary sepsis-induced acute respiratory distress syndrome may be more susceptible to degradation of the eGC than those with pulmonary sepsis.^[30]^

#### 3.2.3. Alterations in circulating cells and formation of microthrombi.

Sepsis can trigger the release of nitric oxide, a potent vasodilator. As a result, its release can alter the blood flow within the microcirculatory system.^[26]^ Moreover, sepsis can significantly alter blood rheology through mechanisms such as endothelial cell damage, cellular dehydration, and adenosine depletion. This results in diminished deformability of red blood cells, rendering them incapable of traversing the microcirculation smoothly. Furthermore, the increased activation and adhesion of leukocytes and platelets can lead to vascular inflammatory response and promote the formation of microthrombi, which can obstruct blood flow at various microcirculatory levels.^[31]^ Consequently, even if macroscopic hemodynamic abnormalities are resolved, the microthrombi may persist, potentially leading to irreversible damage to the microcirculatory system and organs.

### 3.3. Microcirculatory monitoring methods

Physical assessment, several laboratory, and imaging examination methods have been proposed to monitor microcirculation.

#### 3.3.1. Physical assessment.

Capillary refill time (CRT) is the time it takes for the skin to return to the baseline color after applying pressure to the tip of the index finger. A CRT >2 seconds indicates poor tissue perfusion.^[32]^ As a result, CRT is recommended by clinical guidelines as an additional tool to assess volume status in conjunction with other indicators of perfusion.^[1]^

A large randomized clinical trial has shown that targeted resuscitation using CRT as a guide is associated with lower mortality, treatment intensity, and organ failure rates.^[33]^ Ait-Oufella et al^[34]^ found that CRT was strongly correlated with the sequential organ failure assessment score. Additionally, CRT was found to be a strong predictor of mortality at 14 days after initial resuscitation in septic shock. CRT remained high even after the restoration of the macrocirculatory parameters, suggesting that CRT accurately reflected the microcirculation.

Mottling refers to a patchy skin discoloration typically extending around the knee area and is often used as a visual indicator of abnormal skin perfusion. It is clinically evaluated using the skin mottling score developed by Ait-Oufella et al^[35,36]^ This scoring system assigns a numerical score ranging from 0 to 5, with higher scores indicating a greater extent of mottling or skin discoloration around the knee and worse skin perfusion (Table 1).

**Table 1:** Skin mottling score (SMS).

Score	Skin discoloration
0	No mottling
1	Small mottling area (coin size) localized to the center of the knee
2	Moderate mottling area not exceeding the superior edge of the kneecap
3	Mild mottling area not exceeding the middle thigh
4	Severe mottling area not exceeding the fold of the groin
5	Extremely severe mottling area extends the fold of the groin

Based on the extension of the mottling area centered around the knees, the score ranged from 0 to 5. The higher scores, the poorer skin perfusion.

However, conduction skin assessments have several limitations. First of all, the normal values can vary widely between different individuals. Moreover, skin color assessments are prone to observer variations. These measurements can also be affected by ambient temperature and pressure duration. A chronometer could be used to obtain an objective score. However, the assessment still relies on the expertise of an experienced intensive care unit physician.^[7,37]^ As a result, skin assessments can not be used on their own to guide clinical resuscitation.

#### 3.3.2. Laboratory analysis.

Lactate is a byproduct of anaerobic respiration. Studies have shown that serum lactate levels are often elevated following tissue hypoperfusion and cellular hypoxia. Lee et al^[38]^ conducted a retrospective cohort study involving sepsis patients with an initial lactate level of ≥ 2 mmol/L. Their findings revealed that nonsurvivors exhibited significantly higher 6-hour lactate levels and clearance rates compared to survivors. Moreover, after adjusting for confounding factors, both the 6-hour lactate levels and 6-hour lactate clearance rates were associated with 30-day mortality in patients with sepsis. However, apart from hypoxia, several factors can lead to elevated serum lactate levels in the presence of severe microcirculatory disturbances,^[39]^ including the release of inflammatory markers, adrenergic-induced enhancements in aerobic glycolysis, reduced hepatic clearance of lactate, heightened visceral local glycolysis, and the acceleration of the glucose-lactate cycle due to insulin resistance.^[40–42]^ In a rabbit model of endotoxic shock, Zhang et al^[43]^ observed that lactate levels changed within 60 minutes after the induction of tissue damage with LPS infusion. However, alterations in the microcirculatory parameters occurred 30 minutes after the completion of LPS infusion. These findings indicate that variation in lactate lagged behind the onset of microcirculation damage.^[43]^

Other laboratory indices, such as central venous oxygen saturation, the venous-arterial partial pressure difference of carbon dioxide, and central venous-to-arterial carbon dioxide difference combined with arterial-to-venous oxygen content difference (Pcv-aCO2/Ca-cvO2), were also correlated with the microcirculatory perfusion rate.^[44–46]^ These information can assist doctors in understanding the appropriate timing for initiating early fluid resuscitation.

#### 3.3.3. Imaging.

Orthogonal polarized spectroscopic imaging (OPS) involves the use of handheld live microscopes, polarized light, and an orthogonal polarizing mirror to visualize the microcirculation. Specialized software is then used to measure vessel diameters and blood flow velocities in biological tissues.^[47]^ A variation of OPS involves the use of side-flow dark-field imaging (SDF). The advantage of this technique is that it offers a higher resolution and thus facilitates the assessment of erythrocytes and leukocytes within the microcirculation.^[48]^ However, both OPS and SDF images are time-consuming to acquire and analyze. As a result, these techniques are now being replaced with third-generation handheld microscopes based on incident dark field imaging technology, which makes use of high-resolution imaging sensors to capture high-quality images and specialized software to assess the images automatically. Compared to SDF, incident dark field provides superior sublingual microcirculation images and a better assessment of the capillary density.^[49]^

Several other noninvasive monitoring methods have been validated in the assessment of organ microcirculation. Near-infrared spectroscopy is a technique used to assess tissue oxygenation levels by analyzing the distinct absorption spectra of oxygenated and deoxygenated hemoglobin. This noninvasive method is particularly useful for examining regions with minimal adipose tissue, such as the brain and thenar muscles.^[50]^ Nevertheless, it is crucial to take into account various factors that may influence the accuracy of near-infrared spectroscopy measurements, including abnormal local vascular tone resulting from exposure to cold environments, variations in skin pigmentation, and the physiological condition of the patient. Laser Doppler flowmetry and laser speckle contrast imaging share similar underlying principle.^[51]^ Both techniques utilize laser light to induce wavelength changes and generate distinct speckle patterns on stationary tissues and flowing blood cells. The intensity and frequency of these changes are directly linked to the quantity and velocity of the moving blood cells, enabling the visualization of microcirculatory blood flow perfusion. However, laser Doppler flowmetry is constrained by its lower temporal and spatial resolution, which limits its ability to provide clear imaging. On the other hand, laser speckle contrast imaging has lower penetration capabilities and encounters challenges when assessing perfusion in deep organ tissues.^[52]^

The field of bedside imaging technology is continuously advancing, with 1 notable development being contrast-enhanced ultrasound. This technique allows for the real-time observation of microcirculation changes at the patient bedside.^[53]^ By utilizing ultrasound, it is possible to track the movement of contrast agents through the capillaries and visualize tissue microcirculation. Although animal experimental models have shown promising results, the clinical application of contrast-enhanced ultrasound, particularly in intensive care settings, is still in its early stages of development.^[54]^

These monitoring methods are susceptible to external factors such as environmental conditions, temperature fluctuations, technical constraints, and individual physiological variations. As a result, the clinical utility of all these monitoring tools is still limited.^[41,51,55]^ The transition of microcirculation monitoring from a research tool to a bedside monitoring technique utilized by clinical practitioners remains a forthcoming challenge.

#### 3.3.4. Sites used to assess microcirculation.

Various factors, such as the nature of the sepsis pathophysiology and the availability of the technology, influence the choice of the body site for the microcirculation assessment. Clinically, the sublingual mucosa is considered the optimal location for assessing microcirculation in critically ill individuals. This preference is based on the fact that the sublingual mucosa shares an embryonic origin with the visceral circulatory system and can be easily evaluated at the patient bedside.^[56,57]^ Numerous studies have shown that alterations in the sublingual microcirculation can surpass alterations in systemic hemodynamic parameters in terms of their predictive accuracy.^[58]^

In a severely infected porcine model, the OPS technique revealed major alterations in both sublingual and intestinal microcirculation.^[59]^ The microcirculatory changes were correlated with the severity of the infection. Therefore, the use of sublingual microcirculation could be used to assess the organism visceral status. However, even when sublingual microcirculation is well perfused, a slight increase in intra-abdominal pressure can significantly impair the intestinal microcirculatory perfusion. Concordance between the sublingual and intestinal microcirculation is observed only when sepsis-induced inflammatory factors extensively damage the mucosa.^[60,61]^ Therefore, in the management of patients with sepsis, multiparametric measurements should be acquired from multiple sites at different time points because any parameter obtained at 1 location is not representative of an abnormality in another location.

## 4. The significance of microcirculation on the treatment intervention

### 4.1. The correlation between macrocirculation and microcirculation

Cardiac output is a key factor in ensuring adequate blood flow and oxygen delivery to organs and tissues. It is usually used as a monitor of the macrocirculatory flow. However, in the early stages of septic shock, cardiac output increases to compensate for the reduced systemic vascular tone. Therefore, optimal resuscitation tasks cannot be achieved by relying solely on macrocirculatory indices such as cardiac index and mean arterial pressure. However, as the disease progresses, the shedding of endothelial cells leads to vascular and myocardial dysfunction. Ospina-Tascon et al^[62]^ showed that if the circulatory failure persists, even if the macrocirculation is restored to normal, the microcirculation may remain impaired.^[46,63,64]^ Ince et al^[65]^ identified 4 factors that could lead to reduced blood flow within the microcirculatory system in critically ill patients, including uneven blood flow distribution, reduced capillary density due to hemodilution or anemia, vasoconstriction or obstruction, and accumulation of tissue edema. In 2020, Pan et al^[32]^ also showed that over-perfusion and increased capillary regurgitant pressure due to high central venous pressure could also lead to microcirculatory disorders. In view of this, Pan et al assessed the oxygen supply and the mean arterial pressure to ensure that tissues receive an adequate oxygen supply and that blood pressure is maintained within a range that supports tissue perfusion, which is essential for preventing damage to the microcirculatory system and preserve vital organ function within the critical care setting.

### 4.2. Fluid resuscitation and microcirculation

Various factors must be taken into account during fluid resuscitation following septic shock, including time, type of fluid, fluid temperature, rate of administration, duration, and volume of intake.^[66,67]^ Studies have shown that the administration of fluids can increase oxygen delivery, increase the microvascular volume, and improve microvascular perfusion by decreasing the blood viscosity and the adhesion of leukocytes to the endothelium.^[68]^ The administration of colloidal fluid was found to be more effective than saline in improving microcirculation. Dubin et al^[69]^ found that septic shock patients treated with early goal-directed therapy and 6% hydroxyethyl starch (HES) 130/0.4 had a higher capillary microvascular flow index, percentage of perfused capillaries, and density of perfused capillaries than those treated with saline. Xu et al^[70]^ found that microcirculation-directed fluid resuscitation required a significantly lower volume of fluid than blood pressure-directed fluid resuscitation. In the early stage of septic shock, <40% of patients are truly fluid-responsive. On the other hand, it is important to emphasize that all fluid administration may lead to fluid overload and can eventually damage the functional capillary density of the microcirculatory system and, ultimately, lead to renal damage.^[71]^

### 4.3. Vasoactive drugs and microcirculation

Vasoactive drugs are often administered to patients suffering from septic shock to ensure adequate perfusion of cardiomyocytes and vital organs. Studies have shown that the administration of norepinephrine can improve microcirculation, reduce fluid requirements, and minimize the risk of volume overload.^[72]^ Using a rate model with persistent hypotension in deep anesthesia, Fan et al^[73]^ found that compared with fluid administration alone, the addition of norepinephrine infusion could reduce the need for fluid intake, improve the oxygenation of the intestinal mucosa and the microcirculation within the terminal seromuscular layer. Conversely, the administration of epinephrine reduced the microcirculatory blood flow.^[72]^ In the absence of microcirculation, vasoactive agents may reduce the hemodynamic coherence by affecting endogenous receptors and altering the vascular smooth muscle tone.^[74]^ Krupičková et al^[75]^ investigated the relationship between subglottic microcirculatory parameters and macroscopic hemodynamic parameters in a porcine model simulating cardiac arrest and cardiopulmonary resuscitation. The deteriorated microcirculatory status was not only associated with systemic hemodynamic segregation but also strongly correlated with poor prognosis.

### 4.4. Mechanical ventilation and microcirculation

Mechanical ventilation is an effective treatment for hypoxic respiratory failure. According to the Surviving Sepsis Campaign guidelines, higher positive end-expiratory pressures (PEEP) are recommended for adult patients with acute respiratory distress syndrome due to moderate-to-severe sepsis.^[2]^ During mechanical ventilation, high levels of PEEP can affect the hemodynamics of the systemic circulatory system by decreasing the venous return and increasing the resistance of the pulmonary circulation. Recent studies have also indicated that elevated levels of PEEP may damage the alveolar microvascular system. This damage to the alveolar microcirculation can persist even after the restoration of the systemic circulation, resulting in a reduction in capillary density and blood flow.^[76]^ Moreover, mechanical ventilation may also affect visceral perfusion, including the blood flow within the hepatic portal vein and mesenteric vasculature.^[77,78]^ However, current research evaluating the impact of mechanical ventilation on local microvascular perfusion is based on controlled laboratory studies. Therefore, further investigations are warranted to understand the impact of mechanical ventilation on microcirculatory perfusion and to develop more effective clinical monitoring methods.

**Figure 2.**
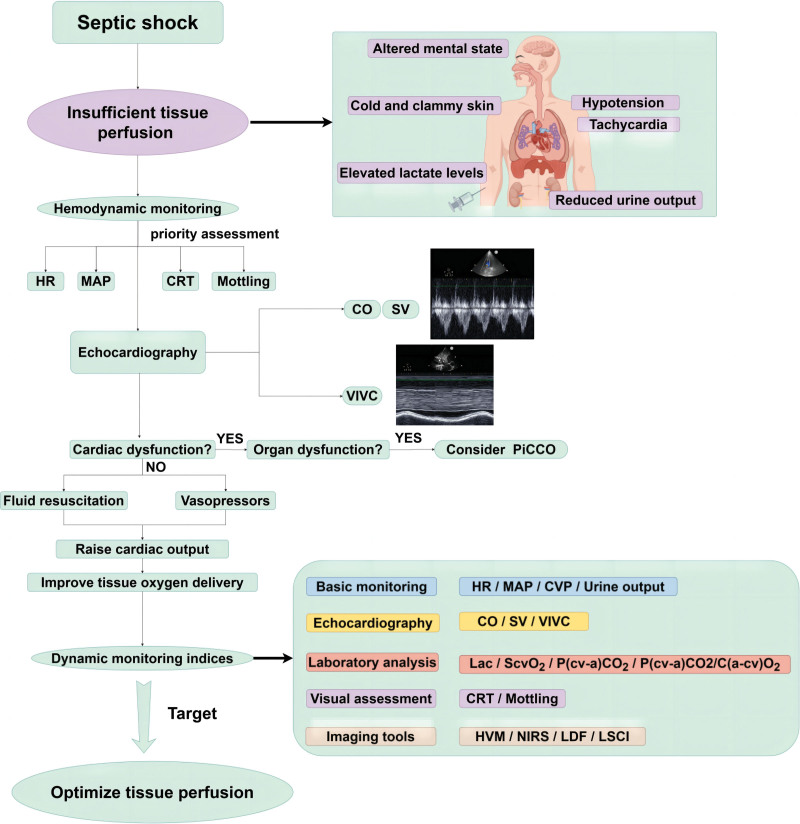
Hemodynamic and physiological monitoring of septic patients. In instances where patients exhibit indications of insufficient tissue perfusion, such as alterations in mental state, reduced urine output, cold and clammy skin, hypotension, tachycardia, elevated lactate levels, among others, it is imperative to prioritize interventions that ensure cardiac output, actively correct volume status, maintain vascular tone. Concurrently, microcirculation monitoring should be regarded as a protective measure, whereby a cautious and precise assessment of microcirculation can facilitate accurate individualized resuscitation and treatment. It is essential to integrate microcirculation monitoring with macrocirculation parameters (by Figdraw). CO = cardiac output, CVP = central venous pressure, HR = heart rate, Lac = lactate, MAP = mean arterial pressure, PiCCO = pulse contour cardiac output, SV = stroke volume, VIVC = variance of inferior vena cava.

Therefore, the complexity of the circulatory condition induced by sepsis poses challenges in devising a targeted treatment approach centered around the microcirculation. However, microcirculation is still necessary for the management of critically ill patients, providing a reliable direction for individualized treatment. Based on previous experience, we have summarized the intervention indicators for hemodynamics at the macro and micro levels, and there may be individual differences in priority order (Fig. 2).

## 5. Conclusions

The normalization of macroscopic hemodynamic parameters in patients with septic shock doesn’t necessarily imply the restoration of microcirculatory perfusion and improved oxygenation. Ongoing and vigilant monitoring of the microcirculatory system is crucial for effectively delivering interventions that enhance tissue perfusion. However, the current tools available for assessing the microcirculatory system are often subjective and not easily implemented in critical care settings. The integration of microcirculation research tools into clinical practice poses a significant challenge for the future. The therapeutic approach to addressing sepsis focuses on enhancing oxygen delivery and tissue metabolism, but excessive treatment interventions may exacerbate microcirculatory decline, particularly in cases of hemodynamic separation. The optimal approach should encompass dynamic, multiparametric, individualized, and continuous monitoring of both the macrocirculation and microcirculation.

## Author contributions

**Conceptualization:** Kun Zhang.

**Supervision:** Hui Wang, Kun Zhang.

**Visualization:** Hui Wang, Hong Ding, Ziyan Wang.

**Writing – original draft:** Hui Wang, Hong Ding.

**Writing – review & editing:** Hui Wang, Kun Zhang.
